# Perioperative redox changes in patients undergoing hepato-pancreatico-biliary cancer surgery

**DOI:** 10.1186/s13741-023-00325-z

**Published:** 2023-07-10

**Authors:** Jia L. Stevens, Helen T. McKenna, Helder Filipe, Laurie Lau, Bernadette O. Fernandez, Andrew J. Murray, Martin Feelisch, Daniel S. Martin

**Affiliations:** 1 Division of Surgery and Interventional Science, Royal Free Hospital, University College London, London, NW3 2QG UK; 2 Royal Free Perioperative Research Group, Department of Anaesthesia, Royal Free Hospital, London, NW3 2QG UK; 3 Peninsula Medical School, University of Plymouth, John Bull Building, Plymouth, PL6 8BU Devon UK; 4 Clinical & Experimental Sciences and Integrative Physiology and Critical Illness Group, Faculty of Medicine, Southampton General Hospital, University of Southampton, Southampton, UK; 5 Department of Physiology, Development and Neuroscience, University of Cambridge, Cambridge, CB2 3EG UK

**Keywords:** Morbidity, Nitrosative stress, Reactive oxygen species, Oxidative stress, Perioperative, Surgery

## Abstract

**Background:**

Tissue injury induces inflammation and the surgical stress response, which are thought to be central to the orchestration of recovery or deterioration after surgery. Enhanced formation of reactive oxygen and nitrogen species accompanies the inflammatory response and triggers separate but integrated reduction/oxidation (redox) pathways that lead to oxidative and/or nitrosative stress (ONS). Quantitative information on ONS in the perioperative period is scarce. This single-centre exploratory study investigated the effects of major surgery on ONS and systemic redox status and their potential associations with postoperative morbidity.

**Methods:**

Blood was collected from 56 patients at baseline, end of surgery (*EoS*) and the first postoperative day (*day-1*). Postoperative morbidity was recorded using the Clavien-Dindo classification and further categorised into minor, moderate and severe. Plasma/serum measures included markers of lipid oxidation (thiobarbituric acid-reactive substances; TBARS, 4-hydroxynonenal; 4-HNE, 8-iso-prostaglandin F_2⍺_; 8-isoprostanes). Total reducing capacity was measured using total free thiols (TFTs) and ferric-reducing ability of plasma (FRAP). Nitric oxide (NO) formation/metabolism was measured using cyclic guanosine monophosphate (cGMP), nitrite, nitrate and total nitroso-species (RxNO). Interleukin-6 (IL-6) and tumour necrosis factor alpha (TNF-⍺) were measured to evaluate inflammation.

**Results:**

Both oxidative stress (TBARS) and nitrosative stress (total nitroso-species) increased from baseline to *EoS* (+14%, *P* = 0.003 and +138%, *P* < 0.001, respectively), along with an increase in overall reducing capacity (+9%, *P* = 0.03) at *EoS* and protein-adjusted total free thiols (+12%, *P* = 0.001) at *day-1* after surgery. Nitrite, nitrate and cGMP concentrations declined concomitantly from baseline to *day-1*. Baseline nitrate was 60% higher in the minor morbidity group compared to severe (*P* = 0.003). The increase in intraoperative TBARS was greater in severe compared to minor morbidity (*P* = 0.01). The decline in intraoperative nitrate was more marked in the minor morbidity group compared to severe (*P* < 0.001), whereas the cGMP decline was greatest in the severe morbidity group (*P* = 0.006).

**Conclusion:**

In patients undergoing major HPB surgery, intraoperative oxidative and nitrosative stress increased, with a concomitant increase in reductive capacity. Baseline nitrate was inversely associated with postoperative morbidity, and the hallmarks of poor postoperative outcome include changes in both oxidative stress and NO metabolism.

**Supplementary Information:**

The online version contains supplementary material available at 10.1186/s13741-023-00325-z.

## Introduction

Improved access to surgery has increased the global burden of postoperative complications and associated long-term health issues (Meara et al. 2015), which is particularly evident after major surgical procedures in a growing population of frail, deconditioned and multimorbid patient group (Pearse et al. 2006). There is a need for a better understanding of the mechanisms that might underpin postoperative complications, in order to develop interventions to reduce harm.

Tissue injury induces sterile inflammation as part of the surgical stress response, both of which are thought to be central to the recovery or deterioration after surgery. Release of reactive oxygen and nitrogen species (ROS and RNS, respectively) accompanies the inflammatory response and triggers separate but integrated pathways that have the potential to lead to cellular disruption and organ dysfunction. Superoxide (O_2_^−^) and nitric oxide (NO) are examples of ROS and RNS, which drive reduction/oxidation (redox) reactions that can lead to a state of oxidative and/or nitrosative stress (ONS) (Stevens et al. 2019). O_2_^−^ in excess leads to the formation of hydrogen peroxide (H_2_O_2_), which can elicit oxidative damage to lipids, proteins and DNA (Kohen and Nyska 2002). Alternatively, O_2_^−^ and NO can combine to form peroxynitrite (ONOO^−^), which supports oxidation, nitrosation and nitration reactions and can promote nitrosative stress (Espey et al. 2000). Reducing compounds are involved in limiting local damage and maintaining overall redox balance. Since NO itself acts as a chain-breaking antioxidant and inhibitor of lipid oxidation (Rubbo et al. 1994), examining the NO metabolic pathways will shed light on its dual role in this context.

There is evidence that surgery can result in oxidative stress, and that the extent of the surgery influences the magnitude of the oxidative response (Aivatidi et al. 2011, Arsalani-Zadeh et al. 2011, Biglioli 2003). Either oxidative stress or NO metabolic markers have been measured in isolation in earlier attempts to demonstrate a link to clinical outcome (Araki et al. 2018, Kaźmierski et al. 2021, Cao et al. 2021, Luo et al. 2021, Satoi et al. 1998, Hirabayashi et al. 2005). Whilst informative, this approach fails to depict the interconnected nature of ROS and RNS within the wider network of redox reactions. In cancer, both oxidative and nitrosative stress play significant roles, yet their effects and inter-relationships in tumour cell biology as well as their responses to chemotherapy are complex (Arfin et al. 2021). Whilst considerable interindividual variability in the extent of reactive oxygen and nitrogen species production by the tumour tissue, affecting circulating biomarker levels, was to be expected, the current study focussed on assessing the effects of major surgery on those levels.

Hepato-pancreatico-biliary (HPB) surgery is known to be associated with a high incidence of postoperative morbidity (Huang et al. 2009, Alexakis et al. 2004). We therefore hypothesised that major HPB surgery would result in increased ROS and RNS formation measured by downstream ONS markers, which in turn would be associated with a worsened postoperative morbidity.

## Methods

We conducted a single-centre, prospective exploratory study of patients undergoing major HPB surgery at the Royal Free Hospital (London, UK). The study was designed and reported according to the Strengthening the Reporting of Observational Studies in Epidemiology (STROBE) guidelines (von Elm et al. 2008).

Ethical approval was obtained from the West London Research Ethics Committee and Human Research Authority [214019]. All patients provided written informed consent prior to surgery.

### Participants

Patients were screened according to the eligibility criteria. Inclusion criteria included the following: major (intra-cavity) inpatient surgery, age ≥ 18 years, planned general anaesthesia and calculated morbidity risk ≥ 40% (Portsmouth Physiological and Operative Severity Score for the enUmeration of Mortality and morbidity — P-POSSUM). Exclusion criteria included the following: mitochondrial disease, emergency surgery, lack of capacity and prisoners. Patients with cancer were not an inclusion criterion at the outset; however, all of the patients who had HPB surgery had a diagnosis of cancer.

### Anaesthetic and surgical techniques

The patients underwent induction of anaesthesia with the intravenous agents fentanyl and propofol, tracheal intubation using a non-depolarising muscle relaxant and maintenance of anaesthesia with a volatile anaesthetic. Neural-axial blockade was used for some patients in the form of epidural or spinal anaesthesia commenced prior to induction of anaesthesia. Patients also received a mixture of opioid and non-opioid analgesia for intraoperative pain relief. A central venous catheter was inserted into the internal jugular vein for venous sampling. The use of vasopressor/inotropic agents and intravenous fluids was commenced based on the physiological and hemodynamic changes observed intraoperatively. After induction of anaesthesia, patients underwent a laparotomy with a subcostal incision and midline extension. The surgical techniques included hepatic and pancreatic resections. The full spectrum of HPB surgery was included because these types of surgery carried theoretically the largest burden of surgical stress conducted at our institution. This included a uniform L-shaped laparotomy, and anaesthetic technique, with varying degrees of resection and vascular occlusion. Including the entire cohort, HPB surgery cohort instead of limiting the study to one modality also aided recruitment of participants.

### Biological sample collection

Samples of venous blood were collected at baseline (after induction of anaesthesia but prior to the first surgical incision), at the end of surgery (*EoS*) after wound closure and 1 day after surgery (*day-1*). The first postoperative day was selected to capture the hyperacute early response to surgery. Samples were immediately placed on ice and centrifuged at 2000 × *g* for 15 min at 4 °C. Plasma and serum samples were divided into aliquots for storage at 80 °C.

### Clinical data collection

Data were collected throughout the perioperative admission. Postoperative complications were graded using the Clavien-Dindo (CD) classification until discharge from hospital (Dindo et al. 2004). For ease of analysis and reduction in multiple group comparisons, five CD groups were merged into three according to the severity of their postoperative complications. The minor complications group was defined as either no complications or a CD score of 1, the moderate complications group defined as CD 2 and the severe complications group included all patients with CD scores > 3. Mortality at 90 days was also determined.

### Blood analysis

The following markers were chosen on the basis of their relationship within the redox network: malondialdehyde (MDA), 4-hydroxynonenal (4-HNE), 8-iso-prostaglandin F_2⍺_ (8-isoprostanes), total free thiols (TFTs), ferric-reducing ability of plasma (FRAP), cyclic guanosine monophosphate (cGMP) and nitrite, nitrate and total nitroso species (RxNO). Interleukin-6 (IL-6) and tumour necrosis factor alpha (TNF-⍺) were measured to evaluate inflammation. Pristine (first thaw from −80 °C) serum or plasma samples were used throughout.

#### Lipid oxidation

Three markers were chosen to assess the extent of lipid oxidation. MDA was measured using the thiobarbituric acid-reactive substance (TBARS) assay by colorimetry (Feldman 2019). The protein adducts of 4-HNE were measured using a competitive ELISA (Elabscience, E-EL-0128), and results were expressed relative to total protein (Spickett 2013). Total 8-isoprostanes were also measured using a competitive ELISA kit following alkaline hydrolysis (Cayman Chemical, 516360).

#### Total reducing capacity

Total reducing capacity was assessed by measuring both thiol-dependent (albumin) and metal-reactive antioxidants. Serum total free thiol levels were determined by a spectrophotometric method using dithionitrobenzoic acid, as previously described Koning et al. 2016. Free thiol concentrations were normalised to overall protein concentration, as serum proteins are the predominant source of thiols (Turell et al. 2013). The ability of plasma to induce one-electron reduction of iron (FRAP assay) was also measured as previously described (Benzie and Strain 1996).

#### Nitric oxide metabolic pathway

The involvement of the NO pathway was investigated using oxidative end products of nitrogen metabolism (nitrite and nitrate), and downstream NO signalling (cGMP), as well as RxNO as a sensitive marker of nitrosative stress. Nitrite and nitrate concentrations were determined using high-performance liquid chromatography (ENO-20, EiCom) (Horscroft et al. 2017); RxNO was quantified using gas-phase chemiluminescence (CLD88, Eco Medics) (Feelisch et al. 2002). cGMP concentrations were determined using a commercially available enzyme immunoassay (R&D systems, KGE003).

#### Inflammatory markers

Serum IL-6 and TNF-α concentrations were measured using a commercially available high sensitivity ELISA kit (R&D systems, HS600C and HSTA00E, respectively).

#### Protein analysis

Serum proteins were measured using a Bradford assay kit (Thermo Scientific).

### Sample size determination

This is an exploratory study, and only a limited number of studies have examined changes in redox status during surgery. We selected two important markers that represented the oxidative and nitrosative arms of the redox network: a marker of plasma lipid oxidation (MDA) and NO metabolism (nitrate). Sample size was calculated using data on these two biomarkers and an *α* of 0.05 and *β* of 0.8 using ClinCalc Sample Size Calculator https://clincalc.com/stats/samplesize.aspx using (detailed in the Supplementary Data section and Supplementary Table 1).

### Statistical analysis

Data were assessed for normality by visual examination of histograms and using the Shapiro-Wilk test. Normally distributed data were presented as mean and standard deviation (SD), and non-normally distributed data were presented as median and interquartile range (IQR). Comparisons of redox measures across the three time-points were performed using repeated measures ANOVA for normally distributed data, and Friedman’s test for multiple related samples, with Wilcoxon rank sum for paired tests for non-normally distributed data. Two snap shots of change in circulating ONS concentration were calculated to reflect the trajectory/dynamics in perioperative redox measures; these include the following: change in intraoperative concentration = *EoS — baseline* and change in overall perioperative concentration = *day-1 — baseline*. These groups were then compared using Kruskal-Wallis for independent multiple comparisons, followed by Mann-Whitney *U* for independent pair-wise comparisons. Pearson’s correlation was performed to demonstrate associations between non-normally distributed continuous data. Missing values were excluded from the analysis. All tests were two-tailed, and *P* < 0.05 was selected as the threshold for statistical significance. In view of the exploratory nature of the study, a decision was made not to correct for multiplicity. Whilst this increased the risk of generation of a type-1 error, it simultaneously reduced the risk of generation of a type-2 error, which was considered important in work of this nature. Statistical analyses were carried out using IBM SPSS version 26 software, and graphs were created using GraphPad Prism 8 software.

## Results

### Clinical information

All 56 patients who underwent surgery had a diagnosis of cancer; the recruitment of participants to this study is summarised in Supplementary Fig. 1. Table 1 and Supplementary Table 2 show the *baseline* participant characteristics. Intraoperatively, 78.6% of patients received a neuraxial block, inotropic support in the form of noradrenaline was used in 89.2% of cases and the median fluid administration intraoperatively was 5.0 l (3.5–6.5 l), where only crystalloid fluid was administered. The median surgical duration was 6.0 h (4.5–7.0 h). The most frequent major intraoperative event that occurred in this cohort was haemorrhage, which happened in 14.3% of cases. Postoperative outcomes are summarised in Table 2. The *baseline* characteristics, type of surgery and intraoperative measurements of the three groups of patients who experienced minor, moderate and severe postoperative complications are summarised in Supplementary Table 4. A breakdown of the complication specifics and the surgical sub-groups can be found in Supplementary Fig. 2 and Supplementary Table 3.

**Table 1:** Participant baseline characteristics

**Characteristics**	***N* (%)**
**Age (years)**^**a**^	67 (60.0–71.7)
**Gender (male, female)**	39, 17 (69.6, 30.4)
**BMI (kg/m**^**2**^**)**^**a**^	25.8 (22.9–28.4)
**Ethnicity**	
**White**	53 (94.6)
**Asian**	2 (3.6)
**Black**	1 (1.8)
**Smoking status**	
**Yes**	5 (8.9)
**No**	31 (55.4)
**Ex-smoker**	20 (35.7)
**Alcohol**	
**Yes**	22 (39.3)
**No**	34 (60.7)
**Consumption (units per week)**^**a**^	2 (2–10)
**ASA score**	
**I**	5 (8.9)
**II**	33 (58.9)
**III**	18 (32.1)
**IV**	0 (0.0)
**Comorbidities**	
**Cardiovascular**	33 (59.8)
**Hypertension**	22 (39.3)
**Ischaemic heart disease**	7 (12.5)
**Heart failure**	0 (0.0)
**Arrhythmia**	4 (7.1)
**Valvular heart disease**	0 (0.0)
**Cerebral vascular disease**	2 (3.6)
**Peripheral vascular disease**	2 (3.6)
**Respiratory**	12 (21.4)
**COPD**	7 (12.5)
**Asthma**	3 (5.4)
**OSA**	1 (1.8)
**Other**	1 (1.8)
**Endocrine and metabolic**	27 (48.2)
**Diabetes**	14 (25.0)
**Hypercholesterolaemia**	8 (14.2)
**Other**	5 (8.9)
**Renal disease**	1 (1.8)
**Rheumatological**	4 (7.1)
**Other systemic disease**	3 (5.4)
**Diagnosis**	
**Pancreatic cancer**	19 (33.9)
**Liver metastasis**	18 (32.1)
**Cholangiocarcinoma**	7 (12.5)
**Hepatocellular carcinoma**	6 (10.7)
**Neuroendocrine tumour**	2 (3.6)
**Ampulla and duodenal cancer**	2 (3.6)
**Other**	2 (3.6)
**Neoadjuvant chemoradiotherapy within the last year**	15 (26.8)

^**a**^Median (IQR). *ASA* score American Society of Anaesthesiology score, *COPD* Chronic obstructive pulmonary disease, *OSA* Obstructive sleep apnoea

**Table 2:** Postoperative outcomes

**Postoperative outcome**	***N* (%)**	
**Clavien Dindo**		Severity category
**No complications**	1 (1.8%)	}Mild: 13 (23.2%)
**I**	12 (21.4%)	
**II**	27 (48.2%)	Moderate: 27 (48.2%)
**IIIa**	3 (5.4%)	}Severe: 16 (28.6%)
**IIIb**	1 (1.8%)	
**IVa**	10 (17.9%)	
**IVb**	1 (1.8%)	
**V**	1 (1.8%)	
**Median (IQR) number of ICU days**	2.0 (1.0–2.8)	/
**Number of ICU readmission**	5 (8.9%)	/
**Median (IQR) number of hospital days**	9.0 (6.2–14.8)	/
**Survival at 90 days**	50 (89.3%)	/

### Differences in circulating redox measures from baseline to day-1 following major surgery

#### Total reducing capacity

**Fig. 1:**
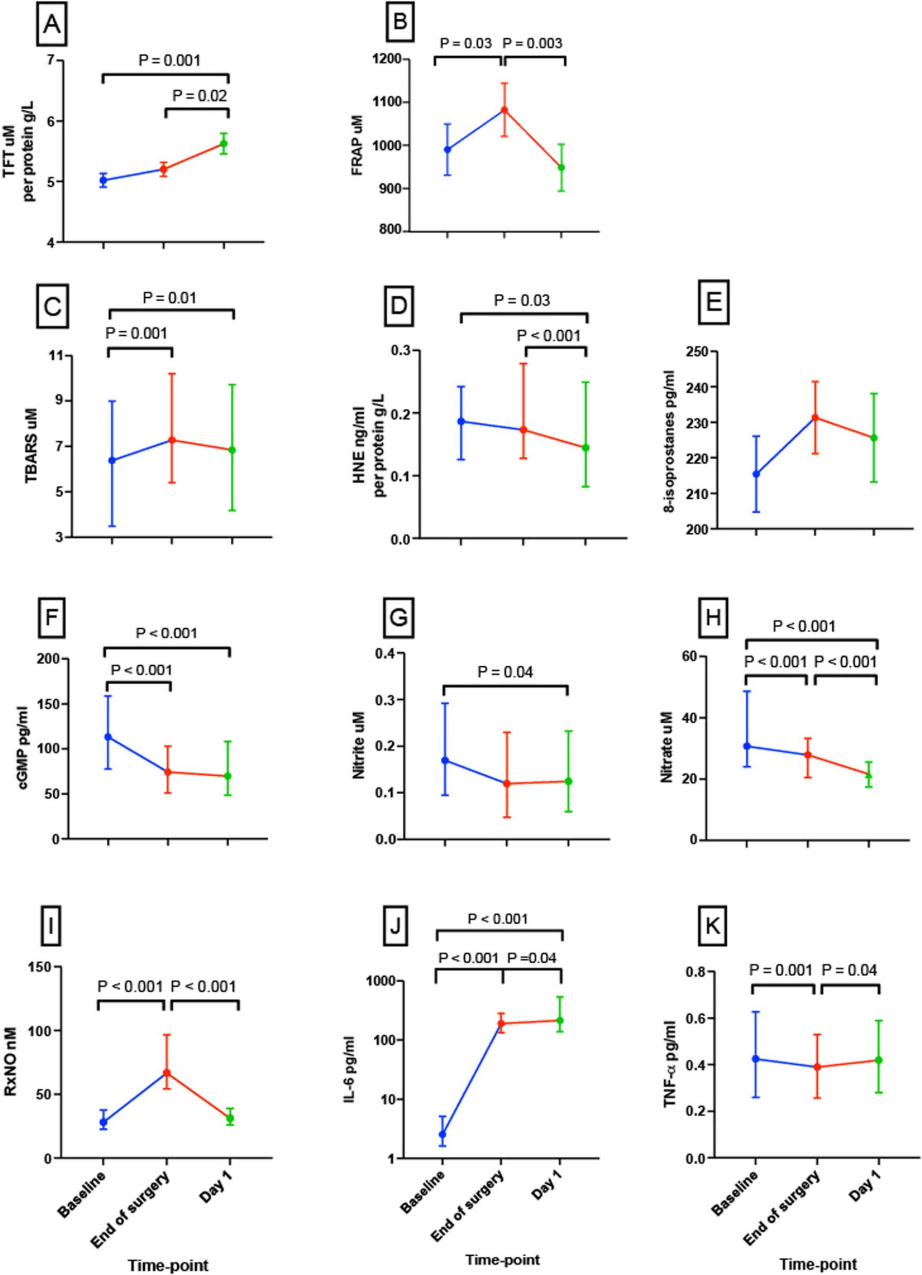
Circulating redox and inflammatory markers measured at *baseline*, *end of surgery* and *day-1* after major surgery. Perioperative changes in **A** TFT adjusted to protein (*n* = 48), **B** FRAP (*n* = 47), **C** TBARS concentration (*n* =4 9), **D** HNE adjusted to protein (*n* = 44), **E** 8-isoprotanes (*n* = 48), **F** cGMP (*n* = 43), **G** nitrite (*n* = 46), **H** nitrate (*n* = 47), **I** RxNO (*n* = 49), **J** IL-6 (*n* = 51), **K** TNF-α (*n* = 51). All panels are presented as median and IQR, except **A**, **B** and **E** are presented as mean and SD, and *y*-axis for **J** is logarithmic

Protein-adjusted TFTs increased by 12.2% from *baseline* to *day-1* following major surgery (*P* = 0.001; Fig. 1A). This increase from *baseline* was not apparent at EoS, but in the time period between EoS and *day-1* after surgery, protein-adjusted TFTs had increased by 8.3% (*P* = 0.02). At EoS, FRAP had increased by 9.3% from *baseline* (*P* = 0.03) but then reverted to *baseline* levels by *day-1* (*P* = 0.003) (Fig. 1B).

#### Lipid oxidation products

The three lipid oxidation products measured in this study demonstrated different trajectories during the acute perioperative period. Plasma TBARS increased by 14.1% at the *EoS* (*P* = 0.003); although there was no change between *EoS* and *day-1* after surgery, the concentration of TBARS at *day-1* remained 7.0% higher than *baseline* (*P* = 0.01, Fig. 1C). In contrast, protein-adjusted 4-HNE adducts decreased by 16.4% from *EoS* to *day-1* (*P* < 0.001), with an overall reduction by 22.5% from *baseline* to *day-1* (*P* = 0.03, Fig. 1D); however, no difference was detected between *baseline* and *EoS*. Meanwhile, the third measure of lipid oxidation, 8-isoprostanes, did not differ significantly across any of the time-points (Fig. 1E).

#### Nitric oxide metabolism

From *baseline*, cGMP concentrations fell 33.0% by the *EoS* (*P* < 0.001), with this difference persisting at *day-1* (*P* < 0.001; Fig. 1F). Similarly, nitrite concentrations decreased by 23.5% on *day-1* after surgery (*P* = 0.04, Fig. 1G). A progressive decrease in nitrate was observed with concentrations falling by 9.3% from *baseline* to the *EoS* (*P* < 0.001) and by a further 22.4% at *day-1* (*P* < 0.001; Fig. 1H). In contrast, RxNO concentrations increased by 138% from *baseline* to the *EoS* (*P* < 0.001) but reverted to *baseline* levels by *day-1* (*P* < 0.001; Fig. 1I).

#### Inflammatory markers

IL-6 increased by 74-fold from *baseline* to *EoS* (*P* < 0.001), with a further, more modest 84% increase on *day-1* after surgery (*P* = 0.04, Fig. 1J). TNF-α decreased by 7.1% from *baseline* to the *EoS* (*P* = 0.001) but reverted back to *baseline* levels by *day-1* (*P* = 0.04, Fig. 1K).

The absolute values of the redox and inflammatory markers at the three time-points are listed in Supplementary Table 5, and perioperative changes protein can be seen in Supplementary Table 6.

### Relationship between baseline circulating redox/inflammatory measures and severity of postoperative morbidity

The *baseline* redox measures in the groups of patients with minor, moderate and severe postoperative morbidity are compared in Table 3. Patients who experienced minor postoperative morbidity had significantly higher *baseline* plasma nitrate concentrations than those who developed moderate or severe postoperative morbidity (*P* < 0.001 and *P* = 0.003, respectively). Both inflammatory markers measured in the study, IL-6 and TNF-α, were significantly higher at *baseline* in the moderate and severe morbidity groups, compared with the minor morbidity group (Table 3).

**Table 3:** Baseline redox and inflammatory markers in groups with minor, moderate and severe postoperative morbidity

	**Minor (*n* = 13)** (IQR)	**Moderate (*n* = 27)** (IQR)	**Severe (*n* = 16)** (IQR)	***p*-value**
**TFT (µM) per protein (g/l)**	5.20 (1.48)	5.12 (0.98)	4.65 (1.13)	0.15
**FRAP (µM)**	953.86 (549.62)	976.23 (523.32)	1084.60 (500.88)	0.40
**TBARS (µM)**	6.19 (2.52)	4.93 (2.33)	5.41 (2.59)	0.97
**HNE (ng/ml) per protein (g/l)**	0.21 (0.20)	0.18 (0.12)	0.18 (0.12)	0.70
**8-isoprostanes (pg/ml)**	251.92 (126.24)	231.99 (87.99)	230.28 (92.06)	0.84
**cGMP (nM)**	88.61 (65.07)	108.41 (69.95)	139.76 (84.20)	0.17
**Nitrite (µM)**	0.18 (0.14)	0.15 (0.21)	0.18 (0.22)	0.83
**Nitrate (µM)**	48.73 (29.19)	27.23 (10.45)	30.45 (10.12)	0.001*
**RxNO (nM)**	29.29 (15.28)	28.58 (14.21)	26.56 (15.66)	0.73
**IL-6 (pg/ml)**	1.60 (1.24)	2.74 (2.92)	4.60 (4.21)	0.006*
**TNF-α (pg/ml)**	0.28 (0.13)	0.45 (0.38)	0.56 (0.44)	0.006*

### Perioperative changes in redox/inflammatory markers in minor, moderate and severe postoperative morbidity groups

**Fig. 2:**
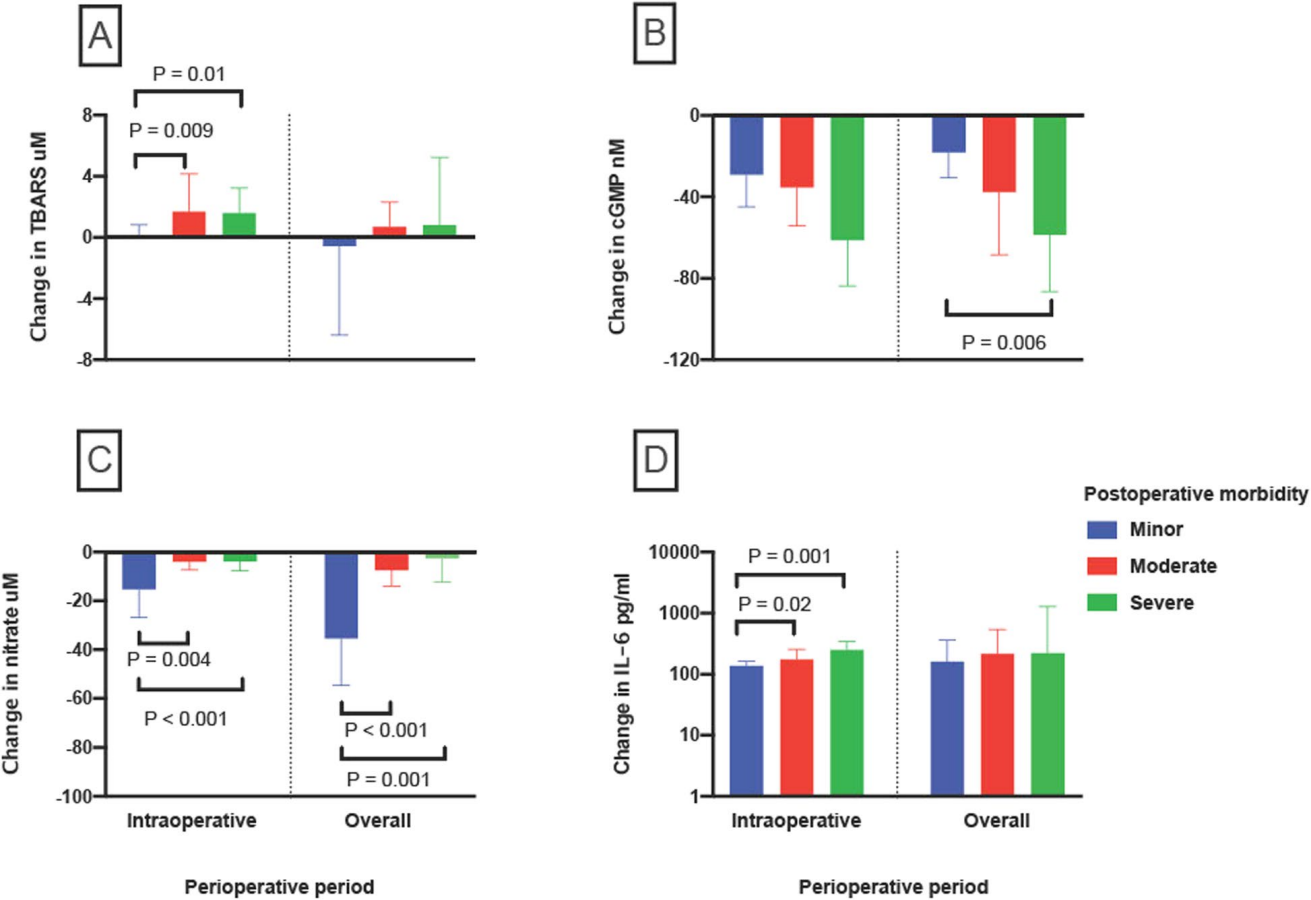
Changes in circulating redox and inflammatory markers in minor, moderate and severe morbidity groups. Comparison of minor, moderate and severe morbidity groups in terms of perioperative changes in the plasma/serum concentration of **A** TBARS, **B** nitrate, **C** cGMP and **D** log_10_ IL-6 (“intraoperative” change calculated as value at EOS minus baseline value; “overall” change calculated as value at day 1 minus baseline value)

The magnitude of absolute intraoperative changes in circulating ONS markers was calculated and compared for minor, moderate and severe postoperative morbidity groups and summarised in Supplementary Table 7. Of note, patients who developed moderate or severe morbidity showed a greater increase in TBARS, in comparison to those with minor morbidity (*P* = 0.02, Fig. 2A). In contrast, patients with minor postoperative morbidity showed a greater fall in nitrate intraoperatively, in comparison to patients who experienced moderate or severe morbidity (*P* = 0.001). Overall, perioperative reduction in nitrate was also greater in the minor morbidity group, in comparison to the groups with moderate or severe morbidity (*P* < 0.001, Fig. 2B). However, a greater overall fall in circulating cGMP concentrations were seen in those with moderate or severe postoperative morbidity, compared to those experiencing minor morbidity (*P* = 0.02, Fig. 2C). Moreover, greater increase in intraoperative IL-6 was witnessed in the severe and moderate morbidity groups compared to the minor group (*P* = 0.001 and *P* = 0.02, respectively, Fig. 2D).

### The effect of duration of surgery, neuraxial blockade and surgical modality on redox and inflammatory markers

Patients who developed severe morbidity underwent significantly longer surgeries (7.0 h (6.0–10.0)) than those who had moderate or minor morbidity (6.0 h (4.0–7.0), *P* = 0.003, and 5.0 h (4.0–6.0), *P* = 0.001, respectively). Therefore, the relationships between duration of surgery and degree of ONS change were explored. Intraoperative change in concentration of TBARS was positively correlated to surgical duration (*r* = 0.318, *P* = 0.03), and overall perioperative change in nitrite concentration was negatively correlated to duration of surgery (*r* = −0.290, *P* = 0.04). No other significant correlations were found.

Comparison of neuraxial blockade and surgical type was not intended at the outset; however, in view of the exploratory nature of this study, these variables were compared against redox and inflammatory markers. Patients who received a neuraxial block demonstrated a greater decline in intraoperative protein-adjusted 4-HNE adduct than those who did not (*P* = 0.003). Conversely, the decline in intraoperative nitrite and nitrate concentrations was greater in patients who did not receive a neuraxial blockade compared to patients with neuraxial block (*P* = 0.03 and *P* = 0.03, respectively). No other differences were detected in other redox or inflammatory markers. In addition, there was no difference in postoperative outcome in patients who received neuraxial blocks compared to those who did not.

Patients underwent three main types of surgery: pancreatic, hepatic and palliative surgery; for a detailed breakdown, see Supplementary Table 3. Biochemical markers were compared between these three groups during the perioperative period. No differences were detected at baseline, and changes in perioperative ONS markers demonstrated some interesting differences in concentrations of TBARS, HNE, RxNO and nitrite between surgical subgroups (see Supplementary Table 8, Supplementary Fig. 3).

## Discussion

The results of this study support the hypothesis that major surgery induces an increase in systemic ONS, with lipid oxidation products (TBARS), markers of nitrosation (RxNO) and inflammation (IL-6) all increasing from *baseline* to the *EoS*. End products of oxidative stress and NO metabolism were linked to a higher incidence of postoperative morbidity. There was also evidence of counter-regulation in the perioperative redox response, with increases in markers of reductive potential, in the form of protein-adjusted TFTs and FRAP after surgery, although these demonstrated different temporal profiles.

Oxidative stress was related to postoperative morbidity, with a greater increase in intraoperative TBARS observed in patients who went on to develop moderate to severe morbidity. In addition, supra-normal levels of *baseline* nitrate, 48.7 µM (normal ranges: 20–40 µM in non-anesthetised individuals) (Lundberg et al. 2008), were observed in patients experiencing minor morbidity. Meanwhile, lower *baseline* inflammatory markers were observed in the same group. Evidence of low-grade inflammation and endothelial dysfunction, as measured by decreased NO availability, has previously been associated with cardiovascular disease (Daiber et al. 2019). The combination of increased *baseline* nitrate and decreased IL-6 may suggest superior vascular health in patients who developed less severe complications.

The majority of published studies describing perioperative redox changes have targeted the oxidative arm alone. Owing to inter-study differences in biomarkers studied, surgical approaches, timings and sites of biological sampling, a direct comparison of our results with those from previous studies is challenging. However, investigations measuring oxidative stress in vascular, general and cardiac surgeries (Aivatidi et al. 2011, Arsalani-Zadeh et al. 2011, Biglioli 2003) have revealed evidence of increased oxidative stress, particularly in the form of MDA, which corresponds with our findings. Similar observations in 8-isoprostanes were made after cardiac and aortic surgery (García-de-la-Asunción et al. 2013, Lindsay et al. 1999). The different perioperative trajectories of the three markers of lipid oxidation measured in this study, MDA, 4-HNE and 8-isoprostanes, may be explained by nonequivalence in their origin and metabolic pathways. The lack of change in 8-isoprostanes in our study may also reflect a less intense surgical stress response in HPB surgery in comparison with cardiopulmonary bypass or aortic surgery. However, it also highlights the challenge of assessing the impact of a complex surgical stress within the redox network by relying on a single biomarker for lipid oxidation to indicate “magnitude of oxidative stress”.

A small number of previous studies have investigated NO metabolism or nitrosative stress in conjunction with oxidative stress, measuring NO using nitrite and nitrate, in combination with TBARS, and typically comparing the effects of open versus laparoscopic surgeries of moderate magnitude. Reported results in these studies are inconsistent, with either no perioperative differences detected in MDA and NO (Ozmen et al. 2002, McHoney et al. 2005) or increased MDA intraoperatively, with reductions in NO during surgery and up to *day-1* thereafter (Zulfikaroglu et al. 2002, Bukan et al. 2004). Data from investigations during major upper gastrointestinal surgery, however, found consistent reductions in NO production (Satoi et al. 1998, Fujioka et al. 2000) supporting our findings. In addition to these, cGMP (a downstream messenger in the NO signalling pathway) was measured in our cohort, which showed a perioperative decline. This might suggest downregulation of the canonical L-arginine-NO pathway during surgery, which could be related to the changes in NO-related chemistry due to enhanced oxidative stress and compromised NO synthase cofactor availability (Madigan and Zuckerbraun 2013). Interestingly, a greater intraoperative decline in nitrate from *baseline* values was a hallmark of patients with the least severe complications, with the converse finding of a greater decline in cGMP in group with severe morbidity, suggesting that alternative NO-producing pathways, e.g. through the sequential reduction of nitrate/nitrite, may have protective roles during the intraoperative phase.

Comparisons of our measurements of protein-adjusted TFTs and FRAP to other published studies is more challenging, owing to the use of non-equivalent antioxidant assays in other work where, in the majority of cases, total antioxidant capacity has been shown to *decrease* in an environment of oxidative stress (Aivatidi et al. 2011, Arsalani-Zadeh et al. 2011). The apparent contradiction may be explained by the assays measuring different antioxidant mechanisms (Rubio et al. 2016). Of note, however, the antioxidant potential of albumin, the predominant source of TFTs, was measured in one study following general and cardiac surgery on day-1, where albumin was found to be in a more reduced state compared to pre-operative levels (Hayakawa et al. 1997), which was similar to our findings. This increased reductive potential could represent an endogenous adaptive system in operation and highlights the risk of using TFTs as a simple marker of systemic oxidative stress.

Studies demonstrating the association of redox measurements with postoperative outcomes have been conducted with either oxidative stress or NO metabolic markers alone. Increased oxidative stress has been associated with postoperative morbidity in cardiac, thoracic and general surgery (Araki et al. 2018, Kaźmierski et al. 2021, Cao et al. 2021, Luo et al. 2021). Specifically, in patients undergoing HPB surgery, increased oxidative stress (using an automated system measuring static oxidation-reduction potential), and inflammation (IL-2 and IL-5) was found in patients with severe complications (Schwarz et al. 2017). Nitrate reduction from *baseline* up to day 3 postoperatively was observed in patients who underwent HPB surgery without complications (Satoi et al. 1998). Although in a separate study measured serum and surgical field nitrate + nitrite were unchanged within the 24-h period, levels rose in the severe complication group between days 3 and 7 (Hirabayashi et al. 2005).

Neuraxial blockade has previously been demonstrated to obtund the surgical stress response (Wang et al. 2019); however, how this relates to perioperative levels of ONS is less clear. In the present study, there is evidence of differences in both oxidative and nitrosative metabolism between patients who have received neuraxial blockade compared to those who have not. A greater decline in protein-adjusted 4-HNE in patients who received neuraxial blockades was observed, and greater reductions in the concentrations of nitrite and nitrate were witnessed in patients who did not receive neuraxial blockade. However, there was no clear evidence of greater ONS or inflammatory markers in patients who did not receive neuraxial blockade, and receiving neuraxial blockade was not associated with better postoperative outcome in this cohort. Therefore, whether neuraxial blockade itself promotes redox alterations that affect postoperative complications remains uncertain.

Previous studies have demonstrated the extent of surgery affecting the degree of oxidative stress (Aivatidi et al. 2011, Arsalani-Zadeh et al. 2011, Biglioli 2003). In our study, we demonstrated weak associations between surgical duration (a surrogate measure for the extent of surgery) and increase in intraoperative TBARS and overall decline in perioperative nitrite. The implication of this is that, ONS may be related to both the magnitude of the surgical insult and the increased morbidity associated with this, although currently there is no standardised clinical way to ascertain the magnitude of surgery, which is generally reflected as a composite of surgical modality/invasiveness, degree of physiological disturbances, blood loss and surgical duration. Comparisons between different HPB surgical subgroups were made as another distinguishing marker of surgical extent or magnitude. The negative findings are perhaps more useful, where the lack of difference in acute inflammatory changes may suggest that the initial magnitude of inflammation is similar between the different surgical groups, with similar baseline exposure to ONS between the surgical modalities. The differences in intraoperative and overall change in lipid oxidation and NO metabolites may be due to ischaemia reperfusion predominating the surgical insult in hepatectomies or as a result of the remaining tumour burden from the unresectable cancer in the palliative surgery group. In addition, the modality of surgery in this cohort was not associated with postoperative morbidity.

### Study limitations

The limited number of time-points may have prevented us from seeing the true ebb and flow of the stress reponse over time. A longitudinal study with multiple time-points particularly at days 3 and 5 would have been desirable, as this is frequently when postoperative complications occur. However, due to resource contraints, it was not possible to collect samples until hospital discharge. In addition, the complications were recorded using the Clavien-Dindo classification; the worst graded complication was used to compare differences in ONS changes, which may not have occurred on *day-1* after surgery.

This was an exploratory study, only powered to detect differences in TBARS and nitrate; therefore, type-2 errors may have occurred for other markers. The acute increase in IL-6 demonstrated the greatest magnitude of response after major HPB surgery, suggesting that sterile inflammation secondary to tissue injury may be one of the main contributors towards ONS. In this pragmatic exploratory study, however, there are other factors at play which may affect redox metabolism, such as underlying patient phenotype, different anaesthetic medications, the changes in macromolecules with antioxidant properties (e.g. bilirubin and albumin) and oxygen use. It was therefore not possible to unpick which of these factors might have had the largest effect on ONS.

### Future studies

From these findings, we might start to characterise phenotypes of individals who are more resilient to the effects of surgery. For example, higher *baseline* nitrate with low systemic inflammation may provide a reflection of superior vascular endothelial health, which can supply more information for anesthests to risk-assess and personalise treatment for patients. By contrast, hallmarks of poor postoperative outcome may include changes in both oxidative stress and NO metabolism, suggesting the interactions between ROS with NO influence the process of recovery. Further mechanistic understanding of how surgical stress affects NO production, metabolism or activation may help us identify new therapeutic targets.

## Conclusion

In patients undergoing major HPB surgery, we have demonstrated measurable early changes in markers of both oxidative and nitrosative stress, including increased lipid peroxide and nitroso species levels, where the former was associated with worse clinical outcomes later during the postoperative phase. The nonuniform and reciprocal changes in oxidant, antioxidant, and metabolic products of ONS may be explained by temporal differences in their origin and chemical reactivity, highlighting the complexities of the redox network.

## Supplementary Information

**Additional file 1: Supplementary Fig. 1.** Study flow diagram (CONSORT diagram). **Supplementary Fig. 2.** Breakdown of postoperative morbidity. I, none/mild; II, moderate; III, severe. **Supplementary Fig. 3.** Changes in circulating redox and inflammatory markers in pancreatic, hepatic and palliative surgical groups. **Supplementary Table 1.** Sample size calculation. **Supplementary Table 2.** Extended baseline characteristics. **Supplementary Table 3.** A breakdown of surgical techniques. **Supplementary Table 4.** Baseline characteristics of minor, moderate and severe postoperative morbidity groups. **Supplementary Table 5.** Circulating redox and inflammatory markers measured at *baseline*, *end of surgery* and *day-1* after major surgery. **Supplementary Table 6.** Protein, osmolality and eGFR measured at *baseline, end of surgery* and* day-1* after major surgery. **Supplementary Table 7.** Changes in intraoperative and overall perioperative redox and inflammatory markers in minor, moderate and severe morbidity groups. **Supplementary Table 8**. Baseline measurements of redox and inflammation compared across types of surgery.

## Data Availability

The datasets used and/or analysed during the current study are available from the corresponding author on reasonable request. Subsets of data are included in the supplementary section as indicated.
