# Supplementary material for: Perioperative redox changes in patients undergoing hepato-pancreatico-biliary cancer surgery
Source: Perioper Med (Lond). 2023 Jul 10;12:35. doi: 10.1186/s13741-023-00325-z (PMC10332014; doi:10.1186/s13741-023-00325-z)
Supplement: Supplementary file 1 — Additional file 1: Supplementary Fig. 1. Study flow diagram (CONSORT diagram). Supplementary Fig. 2. Breakdown of postoperative morbidity. I, none/mild; II, moderate; III, severe. Supplementary Fig. 3. Changes in circulating redox and inflammatory markers in pancreatic, hepatic and palliative surgical groups. Supplementary Table 1. Sample size calculation. Supplementary Table 2. Extended baseline characteristics. Supplementary Table 3. A breakdown of surgical techniques. Supplementary Table 4. Baseline characteristics of minor, moderate and severe postoperative morbidity groups. Supplementary Table 5. Circulating redox and inflammatory markers measured at baseline, end of surgery and day-1 after major surgery. Supplementary Table 6. Protein, osmolality and eGFR measured at baseline, end of surgery and day-1 after major surgery. Supplementary Table 7. Changes in intraoperative and overall perioperative redox and inflammatory markers in minor, moderate and severe morbidity groups. Supplementary Table 8. Baseline measurements of redox and inflammation compared across types of surgery. [file 13741_2023_325_MOESM1_ESM.docx]

# **Supplementary data**

to

**Perioperative redox changes in patients undergoing hepato-pancreatico-biliary cancer surgery**

Jia L Stevens Ph.D^,2^, Helen T McKenna Ph.D^3^, Helder Filipe BSc^2^, Laurie Lau Ph.D^4^, Bernadette O Fernandez Ph.D^4^; Andrew J Murray Ph.D^5^; *Martin Feelisch Ph.D^4^; Daniel S Martin Ph.D^1,3^

1. University College London, Division of Surgery and Interventional Science, Royal Free Hospital, 3rd Floor, Pond Street, London NW3 2QG, UK
2. Royal Free Perioperative Research Group, Department of Anaesthesia, Royal Free Hospital, 3rd Floor, Pond Street, London NW3 2QG, UK
3. Peninsula Medical School, University of Plymouth, John Bull Building, Plymouth, Devon, PL6 8BU, UK.
4. Clinical & Experimental Sciences and Integrative Physiology and Critical Illness Group, Faculty of Medicine, Southampton General Hospital and Institute for Life Sciences, University of Southampton, Southampton, UK
5. Department of Physiology, Development and Neuroscience, University of Cambridge, Cambridge, CB2 3EG, UK

**Supplementary Methods**

## **Sample size calculation**

Table S1 demonstrates the sample size calculations performed, this included a marker of plasma lipid oxidation (MDA) and NO metabolism (nitrate). Sample size was calculated using ClinCalc Sample Size Calculator https://clincalc.com/stats/samplesize.aspx using these parameters (α = 0.05, β = 0.8). Data for MDA ^1^ and nitrate ^2^ were extrapolated from previous HPB surgical studies, using figures at baseline and after surgery. To account for attrition rates, a further 20% was added onto the figure.

**Supplementary Table 1. Sample size calculation**

|  | Mean | SD | % change from mean | Sample size | 20% attrition rate added |
| --- | --- | --- | --- | --- | --- |
| MDA (nmol/mg) | 0.36 | 0.11 | 12.5 | 47 | 56.4 |
| Nitrate (uM) | 30.3 | 3.61 | 5 | 44 | 52.8 |

#### **ELISA, colorimetric assay interpolation and precision of results**

The assays were performed as duplicates or triplicates on the same plate, depending on the availability of sample. volumes The values of assay absorbance were averaged, absorbance against standard concentrations were plotted on a calibration curve using a software (GraphPad Prism 8), with an acceptable r^2^ > 0.99, either using a linear curve or a four-parameter logistic curve, the final assay concentrations were interpolated from the curve.

After completion of the assays, the precision of the results were quality checked, intra-assay variability was assessed using coefficient of variation^[[1]](#footnote-1)^ (CV). This was performed for all assay repeats using Microsoft Excel. The assays were conducted at two separate sites, at University of Southampton, CV < 30% were deemed acceptable from duplicates and for triplicates <15% were acceptable. For all assays performed at University College London, CV of <10% were deemed acceptable. Due to the precious nature of human samples all assays were performed as a single run for all patients, therefore inter-assay variability was not calculated.

The lower limits of detection for the different assays are listed below:

- MDA – 0.26 uM
- 4-HNE – 0.63 ng/ml
- IsoPs – 2.5 pg/ml
- TFT – 0.976 uM
- FRAP – 50 uM
- cGMP – 2.1 pmol/ml
- IL-6 – 0.625 pg/m
- TNF-alpha – 0.156 pg/ml

**Supplementary Figure 1.** Study flow diagram (CONSORT diagram)

135 patients were eligible for recruitment

59 patients consented and enrolled

56 patients underwent surgery. Serum and plasma samples collected at 3 perioperative time-points

56 patients followed-up postoperatively up to discharge, with 90-day survival data collected

3 patients excluded from the study

-1 unfit for surgery

-1 operated in the private sector

-1 cancelled due to ongoing chemotherapy

76 patients refused to take part in the study

56 sets of baseline blood samples were analysed.

51 complete sets of blood samples at time-points 1,2 and 3 were analysed

Enrollment

Intervention

Follow-up

Analysis

**Supplementary Table 2. Extended baseline characteristics**

| Preoperative physiology | Median (IQR) |
| --- | --- |
| Systolic blood pressure (mmHg) | 128 (122-139) |
| Heart rate (bpm) | 72 (63-80) |
| Oxygen saturation (%) | 98 (97-99) |
| Haemoglobin (g/L) | Male 134 (120-144)  Female 126 (121-132) |
| White blood cells (x10^9/L) | 6.5 (5.3-8.1) |
| Platelets (x10^9/L)) | 228 (187-301) |
| Sodium (mmol/L) | 141 (138-143) |
| Potassium (mmol/L) | 4.4 (4.1-4.7) |
| Creatinine (umol/L) | 74 (63-84) |
| Urea (mmol/L) | 4.9 (3.6-5.7) |
| Bilirubin (umol/L) | 8.5 (6-13) |
| Alanine aminotransferase (unit/L) | 25.0 (18.5-49.8) |
| Aspartate aminotransferase (unit/L) | 26.5 (18.3-54.3) |
| Alkaline phosphatase (unit/L) | 104 (72-243) |
| Albumin (g/L) | 43 (39-45) |
| International normalised ratio | 1.0 (1.0-1.1) |
| APTT (s) | 30.4 (29.4-32.9) |

**Supplementary Table 3. A breakdown of surgical techniques**

| Surgical technique | N (%) |
| --- | --- |
| Pancreatic surgery | 18 (32.1) |
| Whipple’s procedure | 13(72.2) |
| Extended pancreatic resection | 5 (27.8) |
| Hepatic resection | 31 (55.4) |
| Wedge resection | 8 (25.8) |
| Extended wedge resection | 6 (19.4) |
| Hepatectomy | 9 (29.0) |
| Extended hepatectomy | 5 (16.1) |
| Other (1^st^ stage ALPPS) | 1 (3.0) |
| Palliative surgery | 7 (12.5) |

**Supplementary Table 4. Baseline characteristics of minor, moderate and severe**

**postoperative morbidity groups**

| **Characteristics** | **Minor** | **Moderate** | **Severe** | **P value** |
| --- | --- | --- | --- | --- |
| **Age (years)** | 68 (67-71) | 65 (67-71) | 70 (66-74) | 0.09 |
| **Female** | 8 (47.1%) | 7 (41.2%) | 2 (11.8%) | 0.01* |
| **Male** | 5 (12.8%) | 20 (51.3%) | 14 (35.9%) |  |
| **BMI** | 23.5  (21.3-26) | 26.1 (21.3-26.0) | 26.0  (23.8-29.0) | 0.09 |
| **Chemotherapy** | 3 (23.1%) | 7 (53.8%) | 3 (23.1%) | 0.86 |
| **ASA**  **I** | 1 (20%) | 3 (60%) | 1 (20%) | 0.15 |
| **II** | 10 (30.3%) | 17 (51.5%) | 6 (18.2%) |  |
| **III** | 2 (11.1%) | 7 (38.9%) | 9 (50%) |  |
| **Diagnosis** |  |  |  |  |
| **Pancreatic cancer** | 6 (31.6%) | 7 (36.8%) | 6 (31.6%) | 0.91 |
| **HCC** | 1 (16.7%) | 3 (50.0%) | 2 (33.3%) |  |
| **Liver metastases** | 3 (16.7%) | 11 (61.1%) | 4 (22.2%) |  |
| **Cholangio-carcinoma** | 1 (14.3%) | 4 (57.1%) | 2 (28.6%) |  |
| **Other** | 2 (33.3%) | 2 (33.3%) | 2 (33.3%) |  |
| **CV diseases** | 5 (18.5%) | 12 (44.4%) | 10 (37.0%) | 0.4 |
| **Respiratory disease** | 3 (30.0%) | 2 (20.0%) | 5 (50.0%) | 0.11 |
| **Diabetes** | 3 (21.4%) | 8 (57.1%) | 3 (21.4%) | 0.79 |
| **Hb (g/L)** | 127 (125-130) | 134 (125-130) | 128 (119-141) | 0.42 |
| **Albumin (g/L)** | 45 (43-48) | 42 (43-48) | 44 (36-46) | 0.11 |
| **Bilirubin (umol/L)** | 8 (6-21) | 8 (6-21) | 8 (6-14) | 0.96 |
| **Type of surgery** |  |  |  |  |
| **Pancreatic** | 3 (16.7%) | 10 (55.6%) | 5 (17.9%) | 0.34 |
| **Hepatic** | 7 (22.6%) | 13 (41.9%) | 11 (35.5%) |  |
| **Palliative** | 3 (42.9%) | 4 (57.1%) | 0 (0%) |  |
| **Operative time (hours)** | 5.0 (4.0-6.0) | 6.0 (4.0-7.0) | 7.0 (6.0-10.0) | 0.0001* |
| **Anesthesia** |  |  |  |  |
| **Propofol (mg)** | 100 (60-148) | 120 (100-150) | 100 (93-158) | 0.20 |
| **Neuraxial block** | 10 | 22 | 12 | 0.87 |
| **None neuraxial block** | 3 | 5 | 4 |  |
| **pH** | 7.39 (7.34-7.41) | 7.35 (7.32-7.39) | 7.33 (7.25-7.40) | 0.08 |
| **Bicarbonate (mmol/L)** | 22.2 (20.8-23.1) | 21.3 (19.6-22.2) | 19.9 (16.5-31.4) | 0.01* |
| **Lactate (mmol/L)** | 1.9 (1.4-2.2) | 2.7 (1.7-3.5) | 4.5 (2.9-6.1) | 0.0001* |
| **Hb (g/L)** | 116.5 (110.3-128) | 117 (104-128) | 84.5 (66.3-116.3) | 0.01* |

**Supplementary Figure 2. Breakdown of postoperative morbidity**

I, none/mild; II, moderate; III, severe

**Supplementary Table 5. Circulating redox and inflammatory markers measured at**

***baseline*, *end of surgery* and *day-1* after major surgery**

| Time-point | | | | |
| --- | --- | --- | --- | --- |
|  | **Baseline** | **End of surgery** | **Day-1** | **P value** |
| TFT (uM) per protein (g/L) | 5.08  (0.98) | 5.37  (1.15) | 5.68  (1.71) | 0.001* |
| FRAP (uM) | 962.53  (584.98) | 1027.03  (627.24) | 980.68  (551.71) | 0.01* |
| TBARS (uM) | 6.38  (5.51) | 7.28  (4.79) | 6.85  (5.54) | 0.001* |
| HNE (ng/ml) per protein (g/L) | 10.10  (6.85) | 7.29  (6.84) | 7.18  (7.05) | 0.001* |
| Isoprostanes  (pg/ml) | 214.44  (104.22) | 237.47 (95.90) | 228.20  (122.16) | 0.28 |
| cGMP (nM) | 113.27 (80.89) | 74.23 (51.81) | 69.68 (59.69) | < 0.001* |
| Nitrite (uM) | 0.17  (0.20) | 0.12  (0.18) | 0.13  (0.17) | 0.04* |
| Nitrate (uM) | 30.74  (24.63) | 27.87  (12.73) | 21.62  (8.18) | < 0.001* |
| RxNO (nM) | 28.04 (15.08) | 66.69 (42.34) | 21.12 (12.85) | < 0.001* |
| IL-6 (pg/ml) | 2.54  (3.49) | 191.23  (151.31) | 215.29  (401.47) | < 0.001* |
| TNF-α (pg/ml) | 0.43  (0.37) | 0.39  (0.28) | 0.42  (0.31) | 0.001* |

**Supplementary Table 6. Protein, osmolality and eGFR measured at *baseline, end of surgery* and *day-1* after major surgery**

| Time-point | | | | |
| --- | --- | --- | --- | --- |
|  | **Baseline** | **End of surgery** | **Day-1** | **P value** |
| Protein (g/L) | 60.53  (0.93) | 45.83  (1.60) | 50.03  (1.20) | < 0.001* |

**Supplementary Table 7. Changes in intraoperative and overall perioperative redox and inflammatory markers in minor, moderate and severe morbidity groups**

| Intraoperative change (*EoS – baseline*) | | | | |
| --- | --- | --- | --- | --- |
|  | **Minor** | **Moderate** | **Severe** | **P value** |
| TFT (uM) per protein (g/L) | 0.46  (1.45) | 0.04  (1.19) | 0.29  (1.65) | 0.53 |
| FRAP (uM) | 166.99  (422.15) | 73.02  (374.49) | -9.72  (310.36) | 0.44 |
| TBARS (uM) | 0.09  (3.40) | 1.71  (4.04) | 1.62  (2.40) | 0.02* |
| HNE (ng/ml) per protein (g/L) | 0.02  (0.17) | 0.02  (0.07) | 0.00  (0.07) | 0.79 |
| IsoPs  (pg/ml) | 15.58  (62.56) | 13.93  (94.99) | 0.56  (98.54) | 0.70 |
| cGMP (nM) | -29.27  (56.89) | -35.48  (66.34) | -61.21  (135.95) | 0.07 |
| Nitrite (uM) | -0.07  (0.11) | 0.00  (-0.03) | -0.04  (0.18) | 0.09 |
| Nitrate (uM) | -15.40  (39.68) | -4.21  (8.32) | -3.89  (9.06) | 0.001* |
| RxNO (nM) | 34.21  (95.31) | 36.56  (-14.11) | 55.43  (58.93) | 0.69 |
| IL-6 (pg/ml) | 137.20  (103.24) | 174.60  (133.20) | 252.90  (145.80) | 0.001* |
| TNF-α (pg/ml) | -0.05  (0.06) | -0.03  (0.10) | -0.11  (0.23) | 3.51 |

| Overall change (*day-1 – baseline*) | | | | |
| --- | --- | --- | --- | --- |
| TFT (uM) per protein g/L | 0.71  (0.99) | 0.74  (1.94) | 0.51  (2.16) | 0.93 |
| FRAP (uM) | 20.23  (355.99) | -14.53  (421.06) | -60.94  622.58 | 0.77 |
| TBARS (uM) | -0.57  (7.99) | 0.71  (2.92) | 0.83  (6.30) | 0.22 |
| HNE (ng/ml) per protein (g/L) | -0.01  (0.07) | -0.03  (0.07) | -0.04  (0.10) | 0.18 |
| Isoprostanes  (pg/ml) | -14.21  (156.64) | 27.80  (102.05) | 3.27  (123.30) | 0.15 |
| cGMP (nM) | -18.34  (34.39) | -37.70  (81.86) | -58.65  (113.24) | 0.02* |
| Nitrite (uM) | -0.03  (0.13) | -0.02  (-0.07) | -0.04  (0.12) | 0.76 |
| Nitrate (uM) | -35.52  (73.77) | -7.53  (16.78) | -2.70  (17.90) | < 0.001* |
| RxNO (nM) | -5.35  (15.49) | 3.54  (-4.91) | 3.48  (34.02) | 0.43 |
| IL-6 (pg/ml) | 162.50  (434.28) | 216.40  (688.80) | 222.90  (1411.50) | 0.32 |
| TNF-α (pg/ml) | -0.03  (0.10) | -0.01  (0.21) | -0.06  (0.21) | 1.57 |

*Change in intraoperative concentration = EoS – baseline*; and *change in overall perioperative concentration = day 1 – baseline*. Morbidity groups compared using Kruskal-Wallis for independent multiple comparisons

**Supplementary Table 8. Baseline measurements of redox and inflammation compared across types of surgery**

|  | Pancreatic | Hepatic | Palliative | Sig. |
| --- | --- | --- | --- | --- |
| Protein adjusted  TFT | 4.71  (0.58) | 5.06  (1.17) | 5.09  (1.26) | 0.67 |
| FRAP (uM) | 919.08  (679.89) | 1012.69  (490.07) | 962.53  (483.51) | 0.45 |
| TBARS (uM) | 5.46  (2.00) | 5.60  (2.68) | 5.10  (2.79) | 0.88 |
| Protein adjusted  HNE | 0.17  (0.14) | 0.19  (0.12) | 0.18  (0.11) | 0.56 |
| Isoprostanes  (pg/ml) | 218.08  (104.31) | 221.18  (122.93) | 210.80  (71.30) | 0.66 |
| cGMP (pg/ml) | 116.35  (54.53) | 112.15  (104.19) | 89.33  (77.24) | 0.83 |
| Nitrite (uM) | 0.24  (0.26) | 0.15  (0.11) | 0.16  0.21) | 0.33 |
| Nitrate (uM) | 36.75  (29.90) | 30.50  (10.12) | 48.63  (34.88) | 0.24 |
| RxNO (nM) | 31.71  (11.96) | 29.10  (19.27) | 25.62  (4.50) | 0.57 |
| IL-6 (pg/ml) | 2.18  (3.36) | 2.72  (3.47) | 4.07  (12.78) | 0.32 |
| TNF(pg/ml) | 0.50  (0.47) | 0.42  (0.38) | 0.35  (0.28) | 0.72 |

**Supplementary Figure 3. Changes in circulating redox and inflammatory markers in pancreatic, hepatic and palliative surgical groups**

**
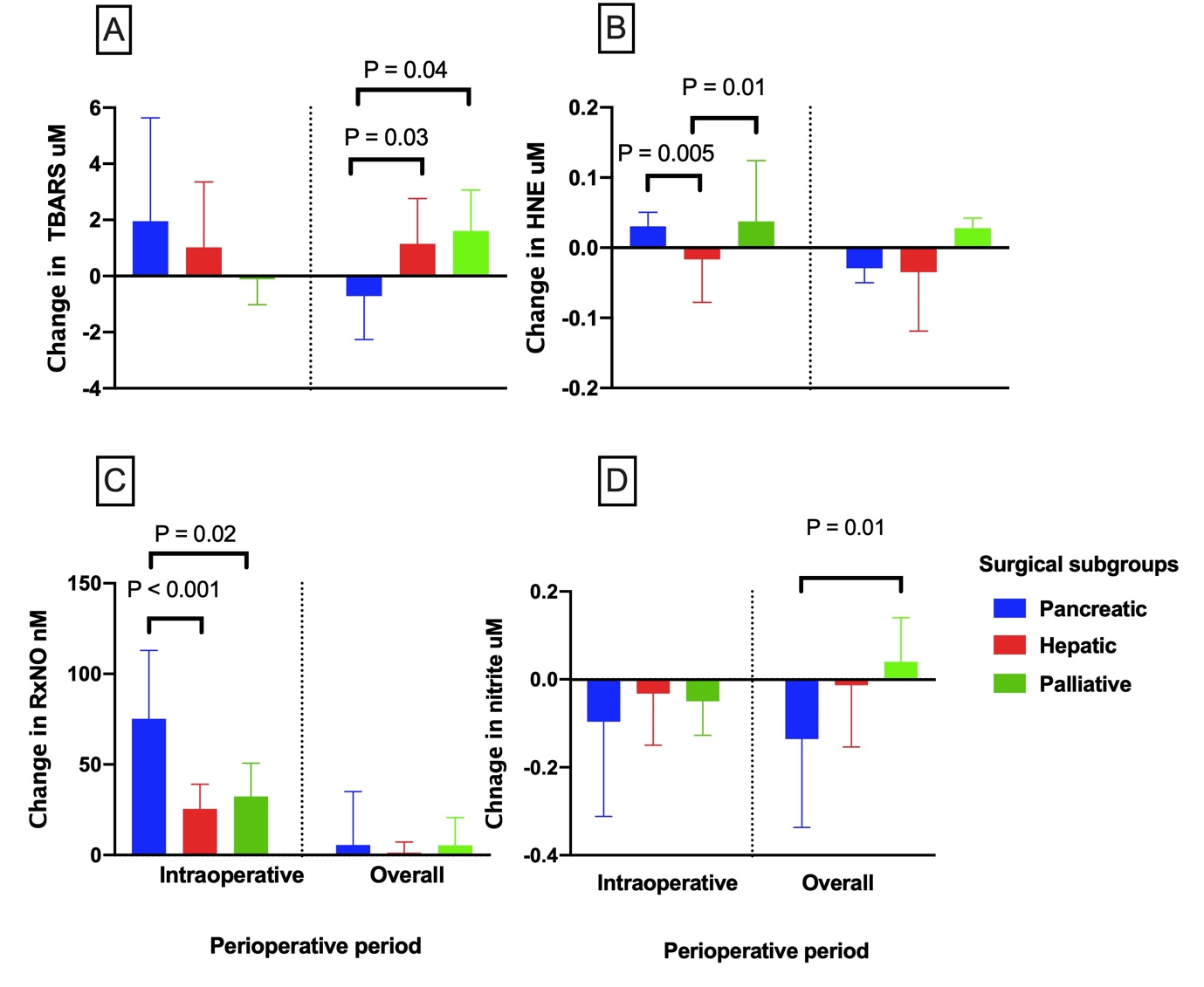
**

*Comparison of pancreatic, hepatic and palliative surgical groups groups in terms of perioperative changes in the plasma/serum concentration of A. TBARS; B. protein adjusted HNE; C: RxNO and D. nitrite. (“Intraoperative” change calculated as value at EOS minus baseline value; “Overall” change calculated as value at Day 1 minus baseline value)*.

Changes in perioperative ONS markers were subsequently compared across surgical subgroups. Differences were found in changes in concentrations of TBARS, HNE, RxNO and nitrite perioperatively. There was a reduction in overall TBARS from *baselin*e to *day-1* in patients who underwent pancreatic surgery compared to hepatic surgery (P = 0.001) and palliative surgery (P < 0.001), where the latter two types of surgeries demonstrated increases in overall TBARS (Figure 3A). Intraoperative protein-adjusted HNE demonstrated significant reduction in the hepatic resection group compared to the pancreatic and palliative surgery group (P = 0.005 and P = 0.01 respectively, Figure 3B). Intraoperative RxNO showed the greatest increase in the pancreatic surgery group compared to hepatic and palliative surgery (P < 0.001 and P = 0.02 respectively, Figure 3C). Lastly, overall nitrite increased in the palliative surgery group which was different from the pancreatic surgery group, where nitrite decreased (P = 0.01, Figure 3D).

**Supplementary references**

1. Witort E, Capaccioli S, Becatti M et al. Albumin Cys34 adducted by acrolein as a marker of oxidative stress in ischemia-reperfusion injury during hepatectomy. Free radical research. 2016;50:831-839.

2. Satoi S, Kamiyama Y, Kitade H et al. Prolonged decreases in plasma nitrate levels at early postoperative phase after hepato-pancreato-biliary surgery. Journal of Laboratory and Clinical Medicine. 1998;131:236-242.

1. Coefficient of variation = (standard deviation/ mean) x100 [↑](#footnote-ref-1)
